# Tunisian sociodemographic profile of elderly patients hospitalized in psychiatry

**DOI:** 10.1192/j.eurpsy.2022.1686

**Published:** 2022-09-01

**Authors:** F. Djemal, N. Messedi, A. Chamseddine, W. Bouattour, F. Charfeddine, L. Aribi, J. Aloulou

**Affiliations:** Hedi Chaker University Hospital Ain road 0.5 Km, Psychiatry B, Sfax, Tunisia

**Keywords:** elderly patients, sociodemographic profile, psychiatry, hospitalization

## Abstract

**Introduction:**

Elderly people have always presented physiological changes and suffered from many diseases. There are few studies focused on this growing particular population, especially with mental pathologies. Thus, psychiatric hospitalization of the elderly population is more frequent nowadays.

**Objectives:**

The aim of this study is to establish the socio-demographic characteristics of elderly patients hospitalized in psychiatry.

**Methods:**

Retrospective and descriptive study over a period of 20 years and 6 months on patients aged over 65 years old hospitalized in the psychiatry “B” department of the Hedi Chaker University hospital in Sfax, Tunisia, for a psychiatric disorder, selected according to the DSM 5 diagnosis criteria.

**Results:**

The number of records identified was 62, out of 4019 patients (15.4%). The mean age of patients was 71.1 years old and the sex ratio (Male / Female) = 0.67. Patients were originally from Sfax in 58.1% and from rural areas in 58.1% of cases. Most of patients (78.4%) were living at least with one member of their family. They were married in 53.2% of cases. The average number of children was 5.21. The majority of patients were illiterate (61.3%) and never had a professional activity in 45.2% of cases. Social coverage concerned 96.8% of our sample.

**Conclusions:**

Elderly patients hospitalized in our department were mainly illiterate, females and living with their family. Despite everything, family involvement in care is still necessary for this category of patients.

**Disclosure:**

No significant relationships.

